# Anti-Inflammatory Activity and Structure-Activity Relationships of Brominated Indoles from a Marine Mollusc

**DOI:** 10.3390/md15050133

**Published:** 2017-05-06

**Authors:** Tarek B. Ahmad, David Rudd, Joshua Smith, Michael Kotiw, Peter Mouatt, Lisa M. Seymour, Lei Liu, Kirsten Benkendorff

**Affiliations:** 1 Marine Ecology Research Centre, School of Environment, Science and Engineering, Southern Cross University, G.P.O. Box 157, Lismore, NSW 2480, Australia; t.ahmad.11@student.scu.edu.au (T.B.A.); david.rudd@scu.edu.au (D.R.); Joshua.Smith@scu.edu.au (J.S.); 2 Centre for Health Sciences Research, University of Southern Queensland, Toowoomba, QLD 4350, Australia; Michael.Kotiw@usq.edu.au (M.K.); lisa.smr@gmail.com (L.M.S.); 3 Analytical Research Laboratory, Southern Cross Plant Science, Southern Cross University, G.P.O. Box 157, Lismore, NSW 2480, Australia; Peter.Mouatt@scu.edu.au (P.M.); ben.liu@scu.edu.au (L.L.)

**Keywords:** marine natural products, inflammation, NO inhibition, Muricidae, isatin, Tyrian purple

## Abstract

Marine molluscs are rich in biologically active natural products that provide new potential sources of anti-inflammatory agents. Here we used bioassay guided fractionation of extracts from the muricid *Dicathais orbita* to identify brominated indoles with anti-inflammatory activity, based on the inhibition of nitric oxide (NO) and tumour necrosis factor α (TNFα) in lipopolysaccharide (LPS) stimulated RAW264.7 macrophages and prostaglandin E2 (PGE2) in calcium ionophore-stimulated 3T3 ccl-92 fibroblasts. Muricid brominated indoles were then compared to a range of synthetic indoles to determine structure-activity relationships. Both hypobranchial gland and egg extracts inhibited the production of NO significantly with IC_50_ of 30.8 and 40 μg/mL, respectively. The hypobranchial gland extract also inhibited the production of TNFα and PGE2 with IC_50_ of 43.03 µg/mL and 34.24 µg/mL, respectively. The purified mono-brominated indole and isatin compounds showed significant inhibitory activity against NO, TNFα, and PGE2, and were more active than dimer indoles and non-brominated isatin. The position of the bromine atom on the isatin benzene ring significantly affected the activity, with 5Br > 6Br > 7Br. The mode of action for the active hypobranchial gland extract, 6-bromoindole, and 6-bromoisatin was further tested by the assessment of the translocation of nuclear factor kappa B (NFκB) in LPS-stimulated RAW264.7 mouse macrophage. The extract (40 µg/mL) significantly inhibited the translocation of NFκB in the LPS-stimulated RAW264.7 macrophages by 48.2%, whereas 40 µg/mL of 6-bromoindole and 6-bromoistain caused a 60.7% and 63.7% reduction in NFκB, respectively. These results identify simple brominated indoles as useful anti-inflammatory drug leads and support the development of extracts from the Australian muricid *D. orbita*, as a new potential natural remedy for the treatment of inflammation.

## 1. Introduction

Inflammation is a complex mechanism involving the activation and deactivation of immune cells in response to stimuli and tightly regulated signalling pathways. If left unchecked, inflammation will result in cellular and tissue damage that can lead to chromic disease [1]. Macrophages play a key role in initiating and maintaining the inflammatory response, and are activated by pathogen associated molecules and cytokines, which stimulate pro-inflammatory signalling pathways [2]. Nuclear factor kappa B (NFκB) has long been considered a prototypical pro-inflammatory signalling molecule, activated by pro-inflammatory cytokines such as tumour necrosis factor α (TNFα) in the presence of viruses, genotoxic agents, or when stimulated by microbial constituents, including lipopolysaccharides (LPS) from bacterial cell walls [3]. Activation of the inhibitory subunit of NFκB, inhibitor of kappa B (IκB) kinase, results in the phosphorylation of IκB proteins bound to NFκB. As a consequence, NFκB translocates to the nucleus, where it regulates the expression of a variety of transcription factors and co-factors, leading to the expression of pro-inflammatory enzymes like cyclooxygenase 2 (COX-2) and inducible nitric oxide synthase (iNOS), which are responsible for stimulating the production of inflammatory signalling molecules [3,4]. TNFα in particular, is considered one of the key inflammatory mediators in endotoxin-induced tissue injury and high levels of TNFα have been correlated with the severity of the inflammatory response [5]. Moreover, NO produced by iNOS is a prominent marker of inflamed tissue and can cause localised toxicity by producing reactive nitrogen oxide species (RNOS) [6] or by reacting directly with proteins or DNA [7]. Furthermore, prostaglandin E2 (PGE2) is produced as a result of the combined enzymatic activity of phospholipase A2 (PLA2) and COX-2, which is also regulated by the activation of NFκB. Therefore, the suppression of excessive production of NO, TNFα, and/or PGE2 in stimulated cells using in vitro assays provides an effective means to evaluate the anti-inflammatory activity of natural products and extracts [8] and intracellular translocation of NFκB can inform the mode of action [3,4]. 

For many centuries, natural products have provided a significant source of leads for drug development. Thirty-one marine compounds have now entered the clinical trials pipeline for drug development, of which seven have been approved by the Food and Drug Administration (FDA-approved) [9]. In particular, a significant number of anti-cancer drug leads have been isolated from marine molluscs [10]. However, only a small proportion of natural products isolated from marine molluscs have been investigated for anti-inflammatory potential [11]. Nevertheless, a lipid extract from the New Zealand Green lipped mussel *Perna canaliculus* has been clinically tested for the treatment of chronic inflammation and is commercially available as a nutraceutical [12]. Preliminary studies on the crude extracts from whelks in the Muricidae family of predatory gastropods indicate that these may also yield secondary metabolites with interesting anti-inflammatory properties. The acetone extracts of *Drupella (Drupa) margariticola* [13] and chloroform extracts from *Purpura persica* significantly reduced carrageenan-induced paw edema in rats, at concentrations well below the toxic limits [14]. Furthermore, lipid extracts from *Rapana venosa* were found to reduce inflammation and improve the healing of skin burns in Wistar rats [15]. These studies support further investigation of the bioactive compounds in muricid molluscs for potential development as anti-inflammatory treatments to replace the conventional steroidal and non-steroidal anti-inflammatory drugs, which have numerous side effects. 

The Muricidae are well-known for their production of the dye molecule Tyrian purple (6,6 dibromoindigo, **1**, Table 1) and related biologically active brominated indoles [16]. These compounds are generated from the precursor indole tyrindoxyl sulfate (**2**, Table 1), which is stored as a salt of the choline ester murexine in the hypobranchial glands and egg masses [17]. The intermediate dye precursors, tyrindoleninone (**3**, Table 1) and 6-bromoistain (**4**, Table 1), have specific anticancer activity, inducing apoptosis in a range of cancer cell lines in vitro [18,19,20,21,22] and DNA damaged cells to prevent colon cancer in vivo [21,23,24]. These bioactive monobrominated indoles dimerise to form the green coloured pigment tyriverdin (**5**, Table 1), the immediate precursor to Tyrian purple, which has no known anticancer activity but it is a potent bacteriostatic agent [25]. Minor pigments of Tyrian purple 6,6′-dibromoindirubin and 6-bromoindirubin (**6**, Table 1) have anti-proliferative properties and are potent inhibitors of glycogen synthase kinase-3 (GSK-3) [26]. Synthetic bromoindirubin derivatives with improved solubility and biological selectivity have been developed [27,28,29], leading to several patents over the last decade [30,31,32].

Depending on the specific mode of action, anti-cancer compounds can often also exhibit anti-inflammatory activity [3,33,34]. In addition to anti-cancer properties, indirubin (**7**, Table 1), a minor pigment in Tyrian purple dye mixtures, prevents the increase of reactive oxygen species (ROS), causing the attenuation of phagocytosis and induction of cell death in the presence of ATP [21,35]. Indirubin derivatives also inhibit the release of the pro-inflammatory cytokines interleukin (IL)-1β and IL-6 in mouse macrophage RAW264.7 cells stimulated with LPS [36]. The anti-cancer monobrominated indoles from Muricidae molluscs are yet to be tested for anti-inflammatory activity. However, the simple indole derivative isatin (**8**, Table 1) inhibits iNOS, COX-2, and TNFα, which results in reduced PGE2 and NO levels in LPS-stimulated mouse macrophages [37]. These isatins, along with some indole derivatives, have been patented for the treatment of inflammation [38,39,40]. Consequently, further investigation of the anti-inflammatory properties of the brominated indoles from Muricidae are warranted. This study aims to investigate the inhibition of NO, TNFα, PGE2, and NFκB translocation by extracts and brominated indoles isolated from hypobranchial gland and egg masses of the Muricidae mollusc *Dicathais orbita*. In addition, the structure activity relationships of a number of brominated indoles were examined, using a suite of synthetic indole derivatives to determine if dimerization and the position of halogenation had any influence on activity.

## 2. Results

### 2.1. Chemical Analysis of the Crude Extracts

**Figure 1:**
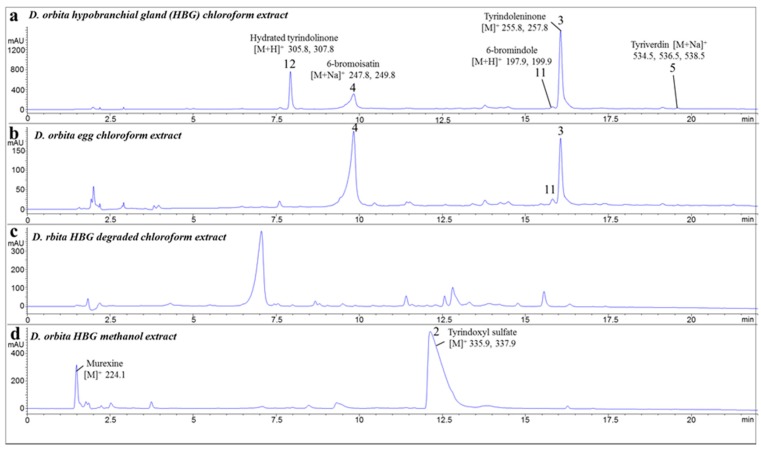
Liquid chromatography mass spectrometry (LC-MS) chromatographs of several extracts from *Dicathais orbita* showing brominated indoles identified by mass spectrometry (molecular ions for Br^79^, Br^81^): (**a**) chloroform extract of the hypobranchial gland; (**b**) chloroform extract of the egg mass; (**c**) degraded chloroform extract of the hypobranchial gland; and (**d**) methanol extract of the hypobranchial gland. Mass spectra for the main brominated indoles are shown in Supplementary Figure S1.

Liquid chromatography mass spectrometry (LC-MS) separations of the chloroform extracts confirmed the presence of brominated indoles typically found in *D. orbita* (Figure 1, Supplementary Figure S1) [17,22,41]. The fresh hypobranchial gland chloroform extract (Figure 1a) and the egg mass chloroform extract (Figure 1b) were dominated by 6-bromoistain (pseudomolecular ion [M + H]^+^ *m*/*z* 226, 228; [M + Na]^+^ *m*/*z* 248, 250, UV λ_max_ 212, 256, 308, and 408 nm) and tyrindoleninone (pseudomolecular ion [M + H]^+^ *m*/*z* 256, 258, UV λ_max_ 236, 248, 274, 352, 402 nm) respectively, and contained smaller amounts of 6-bromoindole (pseudomolecular ion [M + 2H]^+^ *m*/*z* 198, 200, UV λ_max_ 218, 260, 290 nm) and tyriverdin (pseudomolecular ion [M + Na]^+^ *m*/*z* 535, 537, 539; UV λ_max_ 236, 252, 274, 352, 402, and 596 nm) (Figure S1). Another brominated indole, tentatively identified as tyrindolinone (C_10_H_10_BrNOS_2_) [17,20], detected in the hydrated form (pseudomolecular ion [M + H]^+^ 306, 308, UV λ_max_ 224, 254, 338 nm), was more abundant in the hypobranchial gland extracts (Figure 1a). The degraded hypobranchial gland extract did not appear to contain bromoisatin or tyrindoleninone (Figure 1c), and instead was dominated by unidentified compounds that did not produce paired isotopic ions indicative of brominated indoles. Due to the lack of anti-inflammatory activity, this degraded extract was not further characterized. The methanol extract from the hypobranchial gland (Figure 1d) was dominated by the precursor tyrindoxyl sulfate (pseudomolecular ion detected in negative ion mode [M]^−^ *m*/*z* 336, 338, UV λ_max_ 225 and 300 nm) and contained some murexine ([M]^+^ *m*/*z* 224, UV λ_max_ 266 nm (Figure S1)).

**Figure 2:**
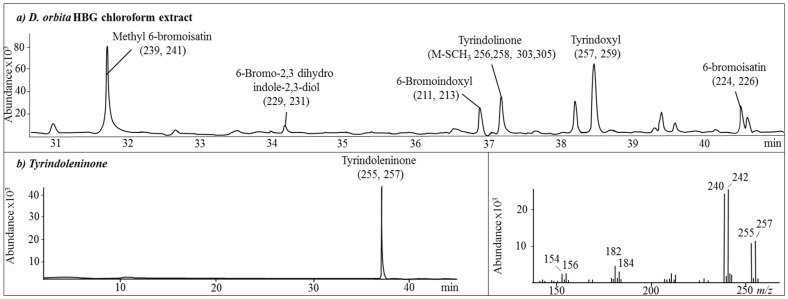
Gas chromatography mass spectrometry (GC-MS) of the chloroform extracts from *Dicathais orbita* showing brominated indoles identified by mass spectrometry (molecular ions for Br^79^, Br^81^). GC-MS chromatograms of the (**a**) hypobranchial gland chloroform extract; (**b**) purified fraction containing tyrindoleninone, with the associated mass spectral fragmentation pattern. The brominated compounds were identified based on characteristic mass isotopic patterns for brominated indoles (Br^79^ and Br^81^), and matched to the NIST02 database. The structures for the main compounds tested are provided in Table 2. The mass spectra for the brominated indoles in the extract are shown in Supplementary Figure S2.

Gas chromatography mass spectrometry (GC-MS) analysis of the hypobranchial gland chloroform extract further confirmed the dominant volatile compounds in the extract were brominated indoles (Figure 2, Supplementary Figure S2). Based on comparison to mass spectral databases (≥98% match), these compounds were identified as methyl 6-bromoisatin, 6-bromo-2,3 dihydro indole-2,3-diol, 6-bromoindoxyl, tyrindoleninone, tyrindoxyl, and 6-bromoisatin (Figure 2a). Tyrindoxyl is a transient compound that readily oxidizes to tyrindoleninone [42,43]. GC-MS confirmed the purity of tyrindoleninone in the isolated orange fraction eluted by silica column chromatography with 20% dichloromethane (DCM) in hexane. 

Tyriverdin is not volatile and is highly insoluble in all solvents. It precipitated out of solution during LC-MS, hence this semi-purified compound could only be tentatively identified using thin layer chromatography (TLC; r.f value = 0.32) and the change from a green to purple colour following exposure to light confirmed that is was mostly likely to be tyriverdin [25].

### 2.2. Cytotoxicity Assays

There was no cytotoxicity observed for any of the *D. orbita* extracts used in this study, at concentrations up to 50 µg/mL, against both RAW264.7 and 3T3 cell lines (Table 2) (cell viability was 100% in all triplicate wells after 24 h of incubation). Therefore, this concentration was chosen as the highest concentration for anti-inflammatory testing. However, at 50 µg/mL, 6-bromoindirubin, tyrindoleninone, 5-bromoisatin, 7-bromoisatin, and 6-bromoindole showed minor toxicity toward RAW264.7 cells after 24 h of incubation with an average % viability of 56.2%, 84.1%, 55.0%, 81.9%, and 66.4%, respectively (Table 2). Only 7-bromoisatin showed slight toxicity at 50 µg/mL against the 3T3 fibroblast cell line after 24 h of incubation, with 80.9% viability when compared to the DMSO-treated wells.

### 2.3. NO Inhibition Assay

**Figure 3:**
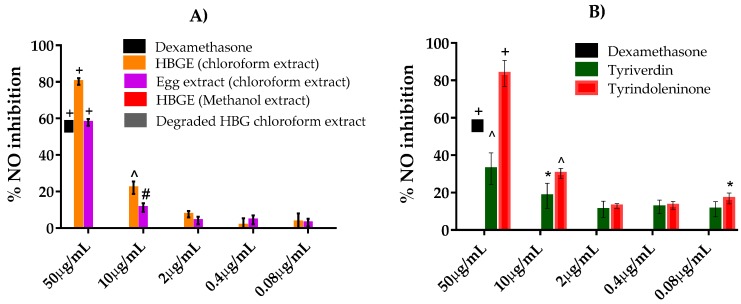

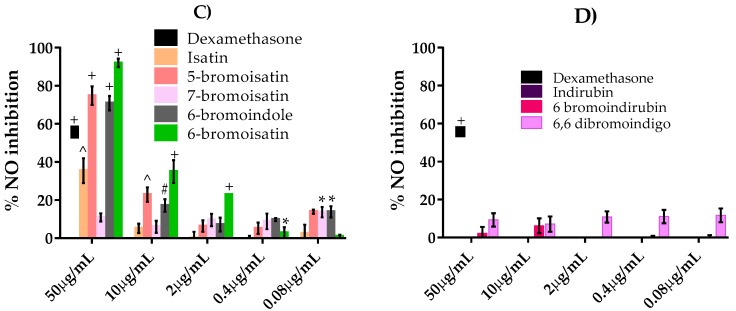
NO inhibition in RAW264.7 mouse macrophages stimulated with lipopolysaccharide (LPS) and treated with extracts from *Dicathais orbita*, purified brominated indoles, and synthetic analogues. Data are expressed as % inhibition of NO production relative to the negative control DMSO: (**A**) extracts from the hypobranchial glands (HBG) and egg capsules; (**B**) the purified natural products tyrindoleninone and tyriverdin; (**C**) the monomer synthetic indoles isatin, 5-bromoisatin, 7-bromoisatin, and 6-bromoindole; (**D**) the dimer synthetic indoles indirubin, 6-bromoindirubin, and 6,6 dibromoindigo. Data shown are means ± SEM from three separate experiments performed in triplicate. The symbols above the bars indicate statistically significant differences in the amount of nitrite in the treatments compared to the DMSO control. * *p* < 0.05, # *p* < 0.01, ^ *p* < 0.001, + *p* < 0.0001 versus untreated, stimulated cells (LPS + DMSO).

The chloroform extracts from *D. orbita* (hypobranchial gland and egg extract) used in this study showed significant inhibition of NO production by LPS-stimulated RAW264.7 cells at concentrations down to 10 µg/mL, in comparison to the DMSO control (*p* < 0.001) (Figure 3A). At the maximum test concentration of 50 µg/mL, the hypobranchial and the egg extract reduced NO production on average by 80% and 57% respectively, compared to the well-known anti-inflammatory drug dexamethasone, which caused 56% inhibition at the working concentration of 2.5 µM (Figure 3A). The methanol extract and the degraded hypobranchial gland chloroform extract, however, showed no inhibition of NO production in the LPS-stimulated RAW264.7 mouse macrophages. The methanol extract was dominated by tyrindoxyl sulfate (Figure 1b), so the lack of NO inhibitory activity in this extracts indicates that this brominated indoxyl-sufate salt also does not have any associated activity (Table 2).

The purified brominated indoles tyrindoleninone and tyriverdin significantly inhibited the production of NO, down to 0.08 μg/mL and 10 μg/mL, respectively (*p* < 0.05, Figure 3B). The IC_50_ for tyrindoleninone was 26.4 μg/mL (103 μM), whereas tyriverdin caused less than 50% reduction in NO at the maximum test concentration (Table 2). The synthetic version of the natural product 6-bromoisatin significantly inhibited NO production down to 0.4 μg/mL (Figure 3B), with an IC_50_ of 27.1 μg/mL (120 μM) (Table 2). The non-brominated compound isatin showed weak NO inhibitory activity causing on average 34% reduction at 50 μg/mL (Figure 3C), with a predicted IC_50_ of 63.3 μg/mL (430 μM). The synthetic 5-bromoisatin had higher activity (Figure 3C) with a predicted IC_50_ of 34.3 μg/mL (151.6 μM, Table 1), whilst 7-bromoisatin showed lower activity, with less than 5% inhibition at 50 μg/mL (Figure 3C). The 6-bromoindole showed similar activity to 5- and 6-bromoisatin (Figure 3C, Table 2). However, all the synthetic dimers including indirubin, 6,6 dibromoindigo, and 6-bromoindirubin showed minimal inhibitory activity (Figure 3D).

### 2.4. TNFα Inhibition

**Figure 4:**
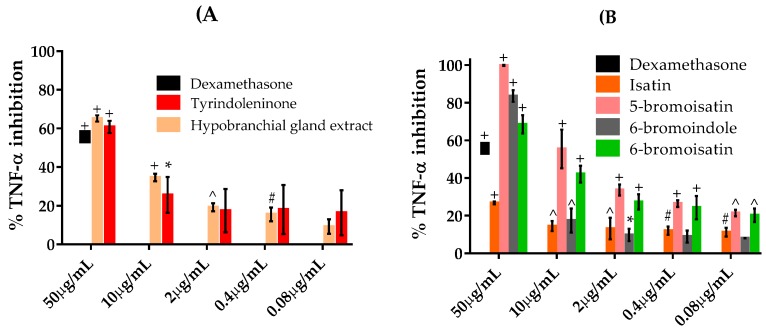
Percent inhibition of TNFα in LPS stimulated RAW264.7 macrophages after treatment with *Dicathais orbita* hypobranchial gland (HBG) extract and associated brominated indoles: (**A**) chloroform extract and tyrindoleninone, purified from the HBG extract; (**B**) synthetic isatin and indole compounds. Data shown are means ± SEM from three separate experiments performed in triplicate. The symbols above the bars indicate statistical significance of the differences in the amount of TNFα in the samples compared to the DMSO control. * *p* < 0.05, # *p* < 0.01, ^ *p* < 0.001, + *p* < 0.0001 versus LPS + DMSO.

The levels of TNFα in the supernatant were measured in LPS-stimulated RAW264.7 cells pre-treated for one hour with the chloroform extract from the hypobranchial gland (HBG), isatin, 5-bromoisatin, 6-bromoisatin, 6-bromoindole, and tyrindoleninone (Figure 4). The HBG extract showed significant inhibition of TNFα, down to 0.4 μg/mL (*p* < 0.01). At 50 μg/mL this extract caused >60% inhibition of TNFα, which was greater than the positive control dexamethasone (Figure 4A). All the brominated indoles tested also showed significant inhibition and for 5-bromoisatin at 50 μg/mL, inhibition reached 100%, with an IC_50_ of 38.05 μM (Table 2). The IC_50_ of the non-brominated indole, isatin, was predicted to be 717.27 μM (above the maximum test concentration), and much less active than the brominated indoles 6-bromoisatin (122.65 μM), 6-bromoindole (150.01 μM), and tyrindoleninone (157.12 μM) (Table 2).

### 2.5. PGE2 Inhibition Assay

**Figure 5:**
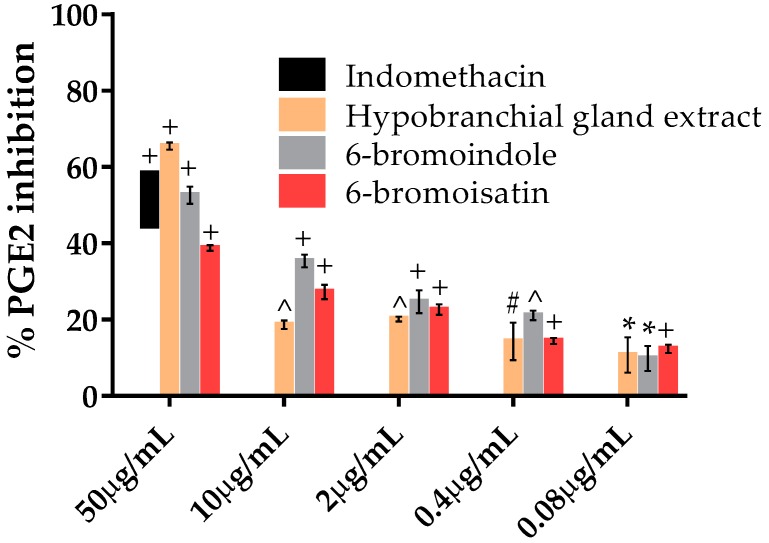
PGE2 inhibition in calcium ionophore stimulated 3T3 ccl-92 fibroblasts after exposure to a chloroform extract from the hypobranchial glands of *Dicathais orbita* and the associated brominated compounds 6-bromoisatin and 6-bromoindole. Data shown are means ± SEM from three separate experiments performed in triplicate. Symbols above the bars indicate statistically significant differences in the amount of PGE2 produced in the samples compared to the DMSO treatment. * *p* < 0.05, # *p* < 0.01, ^ *p* < 0.001, + *p* < 0.0001 versus LPS + DMSO.

The effect of different concentrations of the HBG chloroform extract and the naturally occurring compounds, 6-bromoisatin and 6-bromoindole, on the generation of PGE2 in 3T3 ccl-92 fibroblast was examined. The highest inhibition rate was found using the HBG extract, which inhibited the production of PGE2 by more than 65% at 50 μg/mL (*p* < 0.0001), which is greater than the inhibition caused by indomethacin at the 100 μM concentration (Figure 5). In addition, 6-bromoindole caused a significant dose response for PGE2 inhibition, with significant inhibition compared to the solvent control even at the lowest concentration (0.08 μg/mL) (*p* < 0.05) and IC_50_ of 223.28 μM (Table 2). Similarly, 6-bromoisatin caused a significant dose response with 40% inhibition at 50 μg/mL and just 10% inhibition at 0.08 µg/mL (*p* < 0.0001), with an IC_50_ of 293.02 μM (Table 2).

### 2.6. Assessment of NFκB Translocation

**Figure 6:**
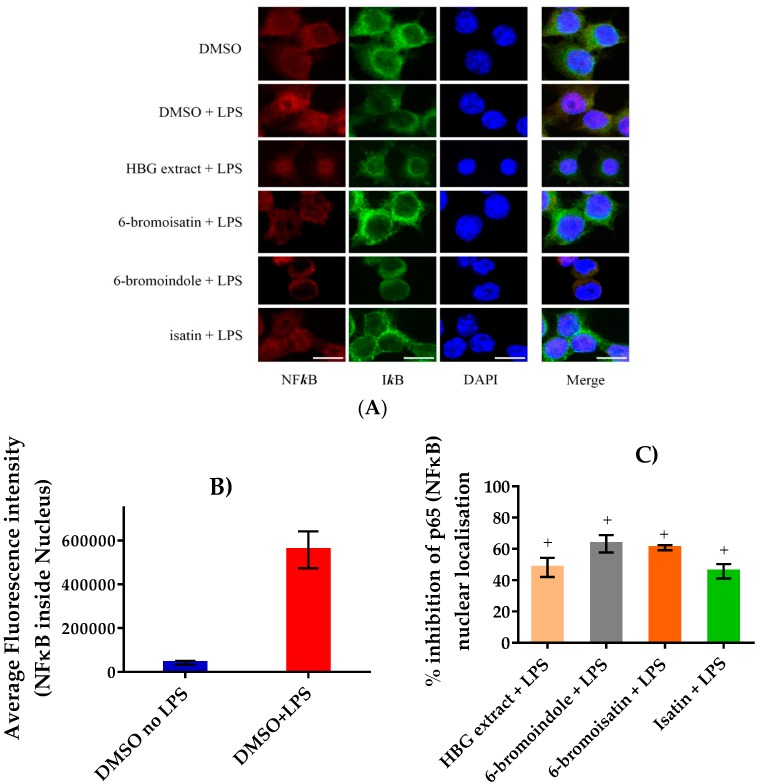
The inhibition of nuclear factor kappa B (NFκB) translocation: (**A**) representative images of the RAW264.7 cells obtained by an Olympus FV i10 confocal microscope showing the effect of each treatment on the translocation of the p65 subunit of the NFκB stained with Alexa fluor 594 (red fluorescence) into the nucleus. The inhibitor subunit was stained with Alexa fluor 488 (green fluorescence) to highlight the inactivated NFκB in the cytoplasm. 4’,6-Diamidino-2-phenylindole (DAPI; blue) was used to stain the nucleus. Scale bar set to 10 μm; (**B**) LPS-induced activation of NFκB in RAW264.7 showing the average intensity of the NFκB fluorescence (red) inside the nucleus; (**C**) mean % NFκB inhibitory activity of the hypobranchial gland (HBG) extract from *Dicathais orbita* and the synthetic compounds 6-bromoisatin, isatin, and 6-bromoindole, based on the reduction of fluorescence intensity relative to the DMSO + LPS stimulated control. All test compounds/extracts were tested at a final concentration of 40 μg/mL. Data shown are mean ± SEM from three separate experiments. “+” = *p* < 0.0001 versus LPS + DMSO. All data were obtained using the image processing and analysis software ImageJ (https://imagej.nih.gov/ij/).

To investigate the mode of action of the active extract and brominated indoles, the ability to inhibit the translocation of the NFκB in LPS-stimulated RAW264.7 was assessed. LPS caused a marked increase in the translocation of NFκB into the nucleus, as indicated by significant increases in the red fluorescence intensity inside the nucleus (Figure 6A,B). The HBG chloroform extract, 6-bromoisatin, isatin, and 6-bromoindole at 40 μg/mL concentration were all found to cause a noticeable inhibition of the translocation of NFκB in the LPS-induced RAW264.7 cells, compared to DMSO (Figure 6A). 6-Bromoindole inhibited the translocation of NFκB by 63.2% on average, whereas 6-bromoisatin, HBG extract, and isatin caused 60.7, 48.2, and 45.7% inhibition, respectively (Figure 6C).

## 3. Discussion

The development of new natural products or drugs that prevent the over-production of NO and pro-inflammatory cytokines, like TNFα and PGE2, has become an important focus of research for the treatment of chronic inflammatory diseases. In this study, crude organic extracts from the Australian marine mollusc *D. orbita* were tested and found to have NO, TNFα, and PGE2 inhibitory activity, but no cytotoxicity at the active concentrations. Consistent with previous studies, the active lipophilic (chloroform) extracts from the hypobranchial glands and egg masses were found to contain the brominated indole intermediate precursors to Tyrian purple dye [17]. Purification of the chloroform extract led to the isolation of tyrindoleninone and tyriverdin, both of which were found to significantly inhibit NO production, along with 6-bromoisatin and several other synthetic monobrominated indoles. By comparison, the synthetic indole dimer pigments and the polar methanol extract containing the salt precursors, tyrindoxyl sulphate, and murexine, were not found to inhibit the production of NO in LPS stimulated RAW cells. This indicates that lipophilic extracts from *D. orbita* containing small brominated indoles have potential use as anti-inflammatory agents, in addition to their previously reported anti-cancer properties [21,22,23,24]. 

A range of synthetic brominated indole derivatives were tested to establish the structure-activity relationships for NO inhibition. Isatin showed mild anti-inflammatory activity, significantly inhibiting the production of NO with IC_50_ at ~339.8 μM and TNFα at concentrations down to 0.5 μM (but with IC_50_ over the maximum test concentration of 50 μg/mL). This activity is generally consistent with that reported by Matheus et al. [37], who found that isatin significantly inhibited LPS and interferon-γ induced NO production in RAW264.7 cells, at even lower concentrations of 10–100 μM, whereas TNFα was inhibited at 100 μM, although they did not establish the minimum effective dose. These researchers also tested a range of simple halogenated isatin derivatives and found that whilst most were active in the test range, the specific activity varied according to the halogen substituent and position. Likewise, our results indicate that the presence and position of bromine on the aromatic ring can affect the activity, with 6-bromoisatin > 5-bromoisatin > isatin > 7-bromoisatin for NO inhibition, whereas for TNFα inhibition, 5-bromoisatin > 6-bromoisatin > isatin. Matheus et al. [37] reported that 4-bromoisatin and 5-iodoisatin did not inhibit NO production or PGE2, whereas three chlorinated isatin derivatives and 5-flouroisatin inhibited NO and PGE2 at similar concentrations to isatin. In the TNFα production assay, however, 5-iodoisatin = 6-chloroisatin > isatin > 5-chloroisatin = 7-chloroisatin > 4-bromoisatin = 4-flouroisatin [37]. These results indicate that the anti-inflammatory activity of isatins depends on both the position and type of halogen substituent, with different patterns for TNFα compared to NO and PGE2 inhibition, suggesting that there may be more than one potential target site or mode of action [37]. Nevertheless, the results from both studies overall suggest that substitution of bromine into the C_5_ and C_6_ position on isatin based compounds effectively increases the anti-inflammatory activity. Previous studies on the anti-cancer activity of isatin derivatives also suggest that bromine substitution on C_5_ or C_6_ can result in increased biological activity [18,44,45].

In addition to the brominated isatin derivatives, we found similar anti-inflammatory activity with synthetic 6-bromoindole and naturally derived 6-brominated indole derivatives. This suggests that the specific functional group in position 2 on the indole ring may not be important for the inhibition of NO, PGE2, or TNFα. Nevertheless, dimerisation of the indoles was found to substantially reduce the activity. The lack of inhibitory activity associated with the brominated indirubin and indigo dimers could be due to their insolubility, as indicated by their calculated log *p* values of >4 (Table 2). Kim and Park [36] synthesised a soluble 3 monoxime derivative of indirubin and demonstrated significant anti-inflammatory activity, including the inhibition of NO and PGE2. However, in our study, the relatively soluble nonbrominated indirubin was also not active and showed a tendency towards stimulating the production of NO in LPS stimulated RAW cells. This result was unexpected given that indirubin is known to inhibit glycogen synthase kinase-3 (GSK3) [45], which promotes anti-inflammatory responses, including LPS induced NO production [46]. Furthermore, indirubin has been identified as an active ingredient in the traditional medicinal plants *Isatis tinctoria* and *Polyglonum tinctorium* used to treat inflammation and has demonstrated in vivo anti-inflammatory properties [47,48,49]. Nevertheless, consistent with our results, indigo and alkaloid fractions obtained from the plant *Indigofera suffruticosa* have been shown to stimulate NO production [50]. This suggests indigo and indirubin may have immunomodulatory activity that depends on the specific cell type and type of stimulation. 

Recent studies have showed that many anti-inflammatory drugs, including dexamethasone, can supress the production of the pro-inflammatory factors by inhibiting the translocation of NFκB [51,52]. Here we provide preliminary evidence that the hypobranchial gland extracts of *D. orbita*, along with isatin, 6-bromoisatin, and 6-bromoindole, exerted anti-inflammatory effects by inhibiting the translocation of NFκB, thus resulting in the inhibition of NO, TNFα, and PGE2 production. Consistent with this, the indirubin analogue indriubin-3-monoxime was found to downregulate NFκB activation, correlating with a reduction of inducible NO synthase (iNOS) and cycloxygenase-2 [36]. Inhibiting the translocation of NFκB prevents the undesirable over production of pro-inflammatory cytokines and NO from the iNOS pathway. Matheus et al. [37] found that isatin and its halogenated derivatives significantly inhibit iNOS and COX-2 protein expression, thus resulting in a significant reduction of NO and PGE2 production. The inhibition of the NFκB translocation signalling pathway is consistent with the duel anti-inflammatory activities and anti-cancer activities of halogenated indole, isatin, and indirubin derivatives from Muricidae molluscs [18,20,21,26,53].

In conclusion, this study supports the nutraceutical potential of extracts from the hypobranchial glands of the Australian muricid *D. orbita* for anti-inflammatory applications. It also confirms that simple brominated isatins have anti-inflammatory activity including the inhibition of NO, PGE2, and TNFα, which are likely to be mediated by the inhibition of NFκB translocation, thus contributing to their potential application and development as anti-inflammatory and anti-cancer agents.

## 4. Materials and Methods

### 4.1. Chemicals and Reagents

*Escherichia coli* LPS (O128:B12, Sigma), sulfanilic acid, *N*-(1-Naphthyl) ethylenediamine (NED), 85% orthophosphoric acid sodium nitrite (NaNO_2_), isatin (**8**, Table 1), 5-bromoisatin (**9**, Table 1), 6-bromoisatin (**4**, Table 1), 7-bromoisatin (**10**, Table 1), 6-bromoindole (**11**, Table 1) were purchased from Sigma-Aldrich (St. Louis, MO, USA). Tyrian purple (6,6 dibromoindigo (**1**, Table 1), indirubin (**7**, Table 1), and 6-bromoindirubin (**6**, Table 1) were obtained from A. L. Skaltsounis (University of Athens). Penicillin–streptomycin solution, Dulbecco’s Modified Eagle’s Medium (DMEM), fetal bovine serum (FBS), sodium pyruvate, and l-glutamine were from Life Technology Australia (Mulgrave, VIC, Australia). RAW264.7 mouse macrophages and 3T3 Swiss albino (ATCC^®^ CCL92™) cell lines were obtained from the American Type Culture Collection (ATCC^®^, Manassas, VA, USA).

### 4.2. Cell Culture

The Murine RAW264.7 and 3T3 fibroblast cell culture were maintained in 10% FBS supplemented with DMEM, 100 μg/L streptomycin, and 100 IU/mL penicillin at 37 °C and 5% CO_2_ atmosphere. Cells were passaged every 48–72 h in a split ratio of 1:10–1:20. Passages used in this study were between passage 7 and passage 25.

### 4.3. Mollusc Collection, Dissection, and Extraction

Medium sized *Dicathais orbita* (40–90 mm shell length) were collected from inter-tidal reefs along the north coast of NSW, Australia, under the Southern Cross University NSW Fisheries exemption permit F89/1171-6.0. The collected snails were kept frozen until needed. In addition, the egg capsules were collected from the same sites during the breeding season in August and stored at −80 °C until needed. The snails were defrosted under running water and the shell was removed by rupturing it carefully using a bench vice. The hypobranchial glands were excised according to Westley and Benkendorff [54] and then weighed on an analytical balance (Mettler-Toledo, Port Melbourne, Australia; precision 0.0001 g).

The extraction of the secondary metabolites from the collected hypobranchial glands (16.06 g) or egg capsules (~12 g) was undertaken according to established procedures [20]. Glands or egg masses were repeatedly soaked for 2 h in solvent (chloroform:methanol, 1:1), and replenished until a clear extract was obtained. The extract was then filtered through Whatman filter paper 1 (90 mm, Sigma-Aldrich) to remove the tissue. The filtered solvent was then decanted into a separating funnel, where the chloroform and the methanol partition were separated with addition of a small amount of MilliQ water. After the two phases formed, both solvent layers were collected separately then evaporated to dryness using a rotary evaporator (Buchi, Flawil, Switzerland). The chloroform partition was kept covered in aluminium foil to protect from photolytic degradation and dried using a vacuum pressure of 474 mbar at 40 °C, then transferred to an amber vial and dried under high purity nitrogen gas before storage at −80 °C. The methanol-water partition was dried using a rotational vacuum concentrator (Alpha-RVC, Martin Christ, Osterode, Germany) until it was totally dry. In a preliminary extraction, *D. orbita* stored for 12 months at −20 °C were dissected and the hypobranchical glands, which had already commenced oxidative and photolytic colour reactions, were used in the extraction. This extract is referred to as the “degraded HBG extract”. To facilitate dissolving this extract in DMSO for anti-inflammatory testing, it was sonicated in a water bath for 30 min, then heated at 60 °C for 10 min, then further sonicated for 10 min and heated for 10 min two more times. For all other extracts and compounds examined, the sonication and heating steps were not required to facilitate solubility in DMSO prior to testing.

### 4.4. Purification of Brominated Natural Products

The crude chloroform extract from the hypobranchial glands was further fractionated using flash silica gel column chromatography in order to purify the main indole compounds. The separation of the compounds based on their colour and polarity by eluting from the silica column with increasingly polar solvents starting with hexane, then DCM, then increasing concentrations of methanol under nitrogen pressure, according to Esmaeelian et al. [22]. Six of the fractions were subject to preliminary bioassay and LC-MS screening and based on these results, further purification focused on the main brominated indoles tyrindoleninone, tyriverdin, and 6-bromoisatin. 

For the separation of tyriverdin from the HBG chloroform extract, the extract was dissolved in hexane, which results in precipitation out of the solution. The precipitate was then centrifuged and the solvent was removed, before washing again in hexane, and repeating the procedure until a clean dried crystaline powder was obtained. To purify tyrindoleninone, the crude hypobranchial chloroform extract was separated using flash column chromatography pressurised with nitrogen gas. The stationary phase consisted of a slurry of silica gel 60 (63–200 μm particle size, 70–230 mesh) mixed with hexane for pouring. Crude extract (426 mg) was loaded onto the column and eluted using a gradient of solvents. Fraction 1 (~120 mL) was eluted with 100% hexane, fraction 2 (~70 mL) was eluted with 20% DCM in hexane, fraction 3 (~230 mL) was collected using 25% DCM in hexane, and fraction 4 with 100% DCM (~250 mL). Fraction 2 containing tyrindoleninone was concentrated in a rotary evaporator at 40 °C and finally dried under N_2_ gas within amber vials for LC-MS analysis and activity testing.

### 4.5. Chemical Analysis of the Extracts and Purified Compounds

Extracts were analysed using an Agilent 1260 Infinity High Performance Liquid Chromatography (HPLC, Santa Clara, CA, USA) system coupled with a 6120 Quadrupole mass spectrometer (MS) according the method outlined in Valles-Regino et al. [41]. The HPLC was undertaken on a C18 reversed phase column (100 × 4.6 mm; Phenomenex Luna, Torrance, CA, USA) using a solvent gradient from 10 to 95% acetonitrile (ACN), both with the addition of 0.005% trifluoroacetic acid (TFA) over 18 min at a flow rate of 0.75 mL/min. The eluent was also monitored using parallel UV/Vis diode-array detection (DAD). The mass spectrometer used electrospray ionisation (ESI) in both positive and negative ion modes, as well as selected ion monitoring for negatively charged brominated indole fragment ions at *m*/*z* 224, 226. Agilent ChemStation was used to analyse the LC-MS data. Brominated indoles were identified by comparison of their retention times and characteristic mass spectra (with major doublet (singly brominated) or triplet (doubly brominated) mass ion clusters separated by 2 mass units for Br^78^ and Br ^81^) as reported in previous studies [41,54].

The chloroform extracts and purified tyrindoleninone were also analysed using Gas chromatography-mass spectrometry (GC-MS) on a Hewlett Packard (HP) 6890 GC coupled to a HP 5973 mass selective detector (MSD; Palo Alto, CA, USA). The GC utilised a column of HP-5MS (Crosslinked 5% PH ME Siloxane) with 30 m × 0.25 mm × 0.25 μm film thickness. The column temperature was controlled by a HP ChemStation programmed from 50 °C (5 min hold) to 250 °C (5 min hold) at 10 °C/min. The injection volume was 0.2 μL using an Agilent 7683 series injector with the injection port (split 1:10) at 250 °C. Helium was used as the carrier gas with the flow rate of 0.7 mL/min, the MS detector source was set at 250 °C and quad at 150 °C, with the transfer line at 280 °C and ion source voltage of 69.9 eV. Data processing was done using HP ChemStation software and the identification of the volatile compounds was based on matches to the library mass spectra and fragmentations patterns (NIST02, WILEY 6, and ESSOILS in house; National Institute of Standards and Technology, Gaithersberg, MD, USA).

### 4.6. Preparation of Extracts and Compounds

All extract/compounds were dissolved in DMSO and tested at 5 different concentrations of 50, 10, 2, 0.4, and 0.08 μg/mL for all anti-inflammatory assays unless otherwise noted. The final concentration of DMSO was 0.35% *v*/*v* for extracts and compounds. All samples were tested in triplicate at each concentration and each assay was independently repeated at least 3 times. The concentrations used for the cytotoxicity assays were 50, 25, 12.5, 6.25. and 3.125 μg/mL.

### 4.7. Cytotoxicity Assay

To check for cytotoxicity of the extracts and compounds against RAW264.7 mouse macrophage and 3T3 ccl-92 mouse fibroblast cell lines, the crystal violet cytotoxicity test was used according to the published protocol [55]. In brief, RAW264.7 cells were seeded with a density of 2 × 10^4^ cells/well in a 96-well plate and then incubated for 18–24 h. The following day the compounds/extracts were added and then incubated for 24 h before the media was aspirated and the cells washed twice in a gentle stream of water. After removing the water, 50 µL of 0.5% crystal violet staining solution were added and the cells were incubated for 20 min at room temperature. The plate was then washed 4 times with water and air dried for 2 h. Finally, 200 μL of methanol was added to each well and incubated for 20 min at room temperature on a rocker. This was followed by measuring the optical density at 570 nm with a plate reader Anthos Zenyth 200rt (Anthos Labtec Instruments, Heerhugowaard, The Netherlands). Chlorambucil was used as a positive control. 

### 4.8. NO Inhibition Assay

The NO inhibition assay was conducted according to published procedures [35,56,57,58,59,60]. RAW264.7 macrophages were seeded in a 96 well plate at a concentration of 6 × 10^5^ cells/mL in colour-free 10% FBS DMEM and incubated at 37 °C in 5% CO_2_ overnight. RAW264.7 macrophages were incubated 1 h with extracts/compounds prior to the addition of LPS. Dexamethasone at a concentration of 2.5 μM was used as a positive control and 0.35% DMSO as a negative control. Then, 20 h after the addition of LPS, the supernatant was collected. The concentration of nitrite in the supernatant was measured using the Greiss reaction by adding an equal volume of the supernatant and Greiss reagent (0.1% NED; 1% sulfanilic acid in 5% orthophosphoric acid) in a 96 well plate. The plates were incubated for 15–20 min in the dark, then read at 550 nm wavelength on an Anthos Zenyth 200rt (Anthos Labtec Instruments). Sodium nitrite was used to prepare a standard curve to quantify the nitrite from the absorbance readings in this assay.

### 4.9. TNF-Alpha Inhibition Assay

The amount of TNFα was quantified in cell culture media using murine TNFα ELISA kits (BD bioscience). In brief, the RAW264.7 mouse macrophages (6 × 10^5^ cells/mL) were stimulated with LPS (100 ng/mL) 1 h after treatment with several concentrations of extracts (0.08–50 μg/mL). After 18 h, the supernatant was collected and TNFα production was measured by reading the absorbance at 450 nm wavelength on an Anthos Zenyth 200rt microplate reader (Anthos Labtech Instruments). Dexamethasone at a concentration of 2.5 μM was used as a positive control, with a negative control of 0.35% DMSO.

### 4.10. PGE2 Inhibition Assay

The levels of PGE2 were measured in cell media using a Cayman Prostaglandin E_2_ Express ELISA Kit (Cayman Chemical, Ann Arbor, MI, USA) according to the manufacturer’s instructions. In brief, the 3T3 ccl-92 fibroblasts (1 × 10^5^ cells/mL) were grown overnight in a 96-well plate and then treated with extracts/compounds 3 h before being stimulated with Calcium ionophore (50 μM). After 20 min, supernatants were collected and PGE2 production was quantified by measuring the absorbance at 405 nm wavelength with an Anthos Zenyth 200rt (Anthos Labtech Instruments). Indomethacin at a concentration of 100 μM was used as a positive control and ~0.5% DMSO as a negative control. 

### 4.11. Assessment of NFκB Translocation

This assay was performed according to the published protocol in Olivera et al. [61]. RAW264.7 mouse macrophages were seeded at 400,000 cells/well in 800 μL in 4-well chambered slides and incubated overnight. The cells were then incubated with the test samples (40 μg/mL HBG extract, 6-bromoisatin, or 6-bromoindole) for 1 h prior to stimulation with LPS (700 ng/mL) for 30 min. After stimulation, the cells were washed with PBS and fixed with 3.7% paraformaldehyde for 15 min. The fixed cells were then washed with PBS three times and permeabilised using 0.2% Triton-X 100 for 10 min, followed by a PBS wash and finally blocked using the 0.1% bovine serum albumin (BSA) for 1 h. The cells were washed with PBS and incubated with rabbit anti-p65 NFκB antibody (1:500) and goat anti-mouse IκB (1:500) overnight at 4 °C. The following day, the cells were washed again three times with PBS and incubated for 1 h with goat-anti-rabbit IgG conjugated with Alexa Fluor 594 (1:1000) and donkey anti-mouse IgG conjugated with Alexa fluor 488 (1:1000) at room temperature. Finally, the cells were washed with PBS then mounted with Prolong mounting media containing DAPI before visualising using the Olympus Flouview FVi10 confocal microscope (Olympus, Tokyo, Japan). Using ImageJ (v1.50i, National Institutes of Health, Bethesda, MD, USA), an outline was drawn around each nucleus by adjusting the colour threshold of DAPI. Mean fluorescence inside the nucleus was measured, along with several adjacent background readings. The total corrected cellular fluorescence (TCCF) was calculated by applying the equation TCCF = integrated density − (area of selected cell × mean fluorescence of background readings) [62]. This TCCF was then used to calculate the %inhibition of the NFκB translocation against the positive control where the cells were treated with LPS in the presence of the Vehicle (0.35% DMSO *v*/*v*) in Microsoft Office Excel 2013 (Redmond, WA, USA).

### 4.12. Statistical Analysis

All data were processed in Microsoft Office Excel 2013 (MS) first to obtain descriptive statistics. One-way Analysis of Variance (ANOVA) followed by Dunnett’s multiple comparisons test was performed using GraphPad Prism version 6.00 for Windows (GraphPad Software, La Jolla, CA, USA, www.graphpad.com), with *p* < 0.05 considered significant. The inhibitory concentration responsible for a 50% reduction in cell viability or production of inflammatory markers (IC_50_) was calculated using the regression analysis, Probit in SPSS v21.

## Figures and Tables

**Table 1:** Chemical structure properties and the source of compounds used in the study.

	Chemical Structure	Molecular Weight ^1^	Log *p* Value ^2^	Synthetic vs. Purified
**1**	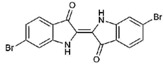	420.06	4.47	Synthetic
**2**	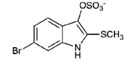	337.18	−0.346	Semi-purified (methanol extract)
**3**	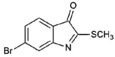	256.12	2.89	Purified
**4**	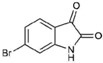	226.03	1.61	Synthetic
**5**	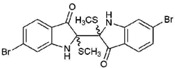	514.25	4.66	Purified
**6**	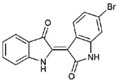	356.18	4.08	Synthetic
**7**	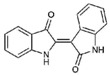	262.27	2.9	Synthetic
**8**	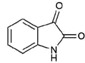	147.13	0.83	Synthetic
**9**	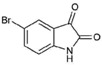	226.03	1.61	Synthetic
**10**	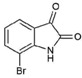	226.03	1.59	Synthetic
**11**	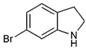	196.05	2.94	Synthetic
**12**	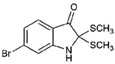	304.22	3.00	Semi-purified (chloroform extract)
**13**	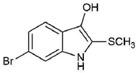	258.13	3.38	Semi-purified (chloroform extract)

^1^ The accurate mass based on the average of the Br^79^ and Br^81^ isotopes. ^2^ The predicted log *p* values were calculated with molecular modelling using Molinspiration© Cheminformatics (2016).

**Table 2:**
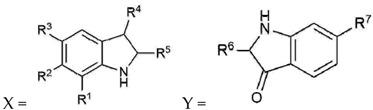
Anti-Inflammatory Inhibitory Concentration 50 (IC_50_) for the brominated indole natural products from *Dicathais orbita* and synthetic analogues. NA = not active at maximum test concentration 50 µg/mL; NT = Not tested.

Compound	R^1^	R^2^	R^3^	R^4^	R^5^	R^6^	R^7^	Cytotoxicity * RAW264.7	Cytotoxicity * 3T3 ccl-92	NO IC_50_ µM	TNFα IC_50_ µM	PGE2 IC_50_ µM
6,6 dibromoindigo (**1**)	H	Br	H	O	Y	X	Br	NA	NA	NA	NT	NT
Tyrindoxyl sulfate (**2**)	H	Br	H	OSO_3_^−^	SCH_3_	-	-	NA	NA	NA	NT	NT
Tyrindoleninone (**3**)	H	Br	H	O	SCH_3_	-	-	>195.22	NA	103	157	NT
6-bromoisatin (**4**)	H	Br	H	O	O	-	-	NA	NA	120	123	>221.21
Tyriverdin (**5**)	H	Br	H	O	SCH_3_, Y	SCH_3_, X	Br	NA	NA	>97.23	NT	NT
6-bromoindirubin (**6**)	H	Br	H	Y	O	X	H	~140.38	NT	NA	NT	NT
Indirubin (**7**)	H	H	H	Y	O	X	H	NA	NA	NA	NT	NT
Isatin (**8**)	H	H	H	O	O	-	-	NA	NA	339.83	>339.83	NT
5-bromoisatin (**9**)	H	H	Br	O	O	-	-	~221.21	NA	152	38	NT
7-bromoisatin (**10**)	Br	H	H	O	O	-	-	>221.21	>221.21	NA	NT	NT
6-bromoindole (**11**)	H	Br	H	H	H			>255.04	NA	187	150	223

* The test concentration (µM) which causes toxicity (reduction in cell viability) toward the cell line after 24 h of incubation; > indicates some activity at the maximum test concentration but with <50% reduction in cell viability or inhibition of the inflammatory markers. ~ indicates close to 50% reduction in viability at the maximum test concentration.

## Supplementary Materials

The following are available online at www.mdpi.com/1660-3397/15/5/133/s1.
