# Supplementary material for: Anti-Inflammatory Activity and Structure-Activity Relationships of Brominated Indoles from a Marine Mollusc
Source: Mar Drugs. 2017 May 6;15(5):133. doi: 10.3390/md15050133 (PMC5450539; doi:10.3390/md15050133)
Supplement: Supplementary file 1 [file marinedrugs-15-00133-s001.pdf]

Supplementary Materials: The following are available online at www.mdpi.com/link

Anti-Inflammatory Activity and Structure- Activity Relationships of Brominated Indoles from a Marine Mollusc

Tarek B. Ahmad[1, 2], David Rudd[1], Joshua Smith[1, 3], Michael Kotiw[2], Peter Mouatt[3], Lisa M. Seymour[2], Lei Liu[3], Kirsten Benkendorff[1*]

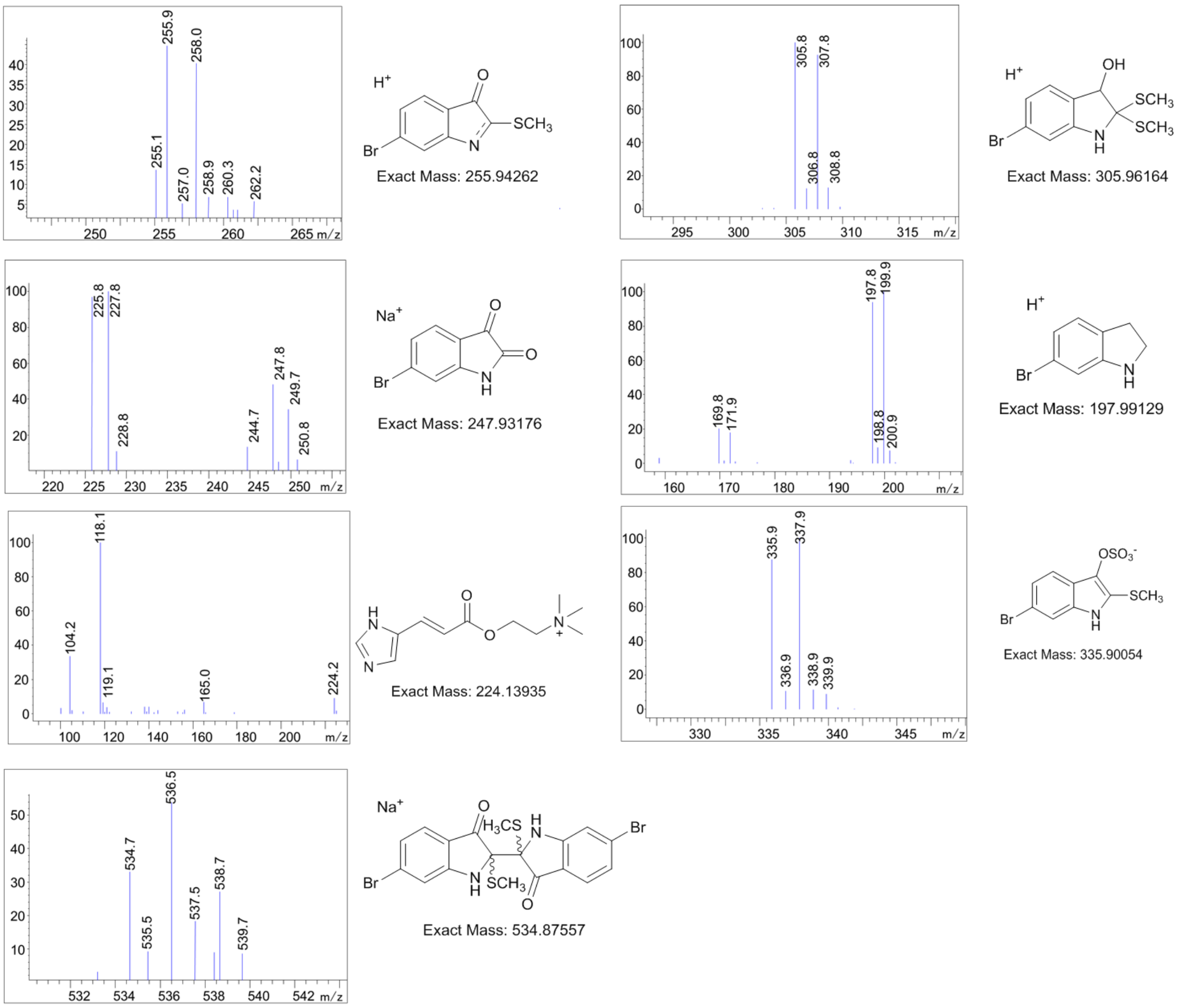

Figure S1: Mass spectral fragmentation patterns of the brominated indoles detected by LC/MS in an extract from the hypobranchial glands of *Dicathais orbita*.

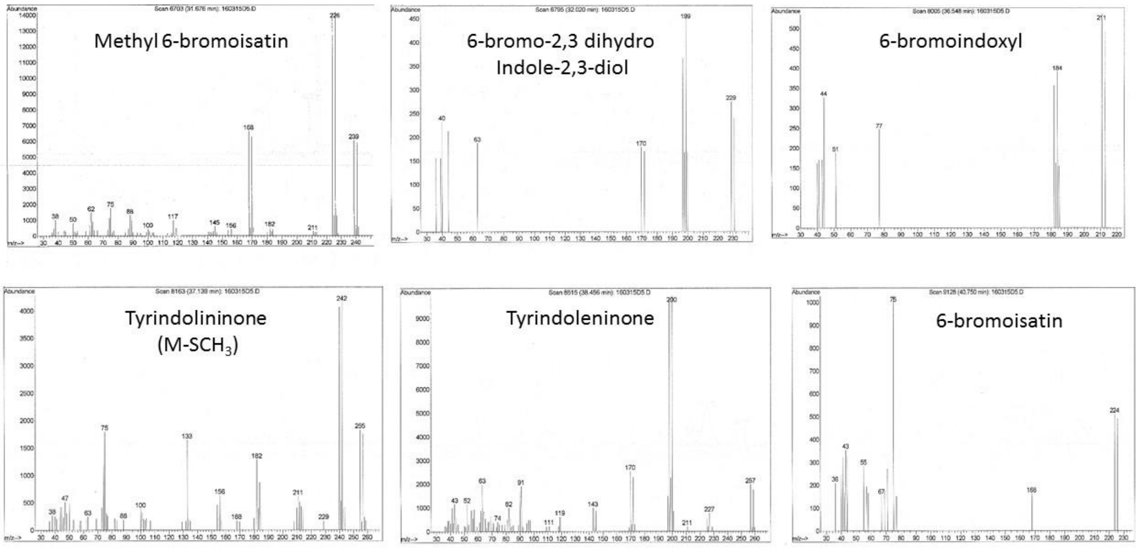

Figure S2: Mass spectral fragmentation patterns of the brominated indoles detected by GC/MS in an extract from the hypobranchial glands of *Dicathais orbita*.
